# Quality Attributes and In Vitro Bioequivalence of Different Brands of Amoxicillin Trihydrate Tablets

**DOI:** 10.3390/pharmaceutics9020018

**Published:** 2017-05-20

**Authors:** Moawia M. Al-Tabakha, Khairi M. S. Fahelelbom, Dana Emad Eddin Obaid, Sadik Sayed

**Affiliations:** 1 Pharmaceutics Unit, College of Pharmacy and Health Sciences, Ajman University, P.O. Box 346, Ajman, UAE; 2 Department of Pharmaceutical Sciences, College of Pharmacy, Al-Ain University of Science and Technology, P.O. Box 64141, Al Ain, UAE; khairi.mustafa@aau.ac.ae (K.M.S.F.); dana.obaid@aau.ac.ae (D.E.E.O.); sadik.sayed@aau.ac.ae (S.S.)

**Keywords:** amoxicillin tablets, bioequivalence, weight variation, friability, chemical content, difference factor, similarity factor, HPLC analysis

## Abstract

Bacterial resistance and antibiotic drug effectiveness can be related to administering generic products with a subtherapeutic dose or poor in vivo drug release. The aim of this study was to investigate whether locally marketed amoxicillin tablets have the required chemical and physical attributes, including in vitro bioequivalence performance. Five generic products (T1, T2, T3, T4, and T5) containing combination of amoxicillin trihydrate and potassium clavulanate as 1 g strength present in immediate release tablets were compared to the reference listed drug product Augmentin^®^ (R) for weight variation, friability, resistance to crushing, and chemical content of amoxicillin. Difference (*f*_1_) and similarity (*f*_2_) factors were calculated to assess in vitro bioequivalence requirements. The tablets from different products have shown compliance with the pharmacopeial requirements of the performed tests. The measured resistance to crushing of tablets did not influence the dissolution time. Three generic products released more than 85% of amoxicillin by the first 15 min as did the reference product and were considered as bioequivalent products. T1 and T4 had *f*_1_ values of 16.5% and 25.4% respectively and their *f*_2_ values were 44.5 and 34.6 respectively, indicating failure to meet in vitro bioequivalence requirements. Tablet formulations can play an important role in achieving bioequivalence. Independent investigations such as this study serve as an important tool to reveal possible inferior or noncompliant products that may find their way to the market.

## 1. Introduction

Amoxicillin, a β-lactam antibiotic, is one of the most widely prescribed antibiotics for a variety of conditions and patient groups [1,2,3]. It is in the list of essential medicines adopted by the World Health Organization (WHO) [4]. Because it is a broad spectrum antibiotic, it has been indicated for a variety of infections including urinary-tract infections, otitis media, sinusitis, pneumonia, salmonellosis, and oral infections [5]. Although the β-lactam drug clavulanateis usually combined with amoxicillin, it is not effective itself as an antibiotic. Its purpose is to overcome amoxicillin resistance in bacteria that secrete β-lactamase and hence has the ability to enhance amoxicillin effectiveness [6].

In 1974, the FDA approved Amoxil as the first brand for amoxicillin, while Augmentin^®^ was the first brand to be approved in 1984 containing a combination of amoxicillin and clavulanate. Both Amoxil and Augmentin^®^ are currently marketed by GlaxoSmithKline (Brentford, U.K.). After their patent expiry, several generic products became available, which are sold at a lower price. From an economical point of view, it is preferable to use generic products, because they are proven to have the same efficacy [7,8,9]. Since generic drug products normally gain marketing approval after documenting to the proper authority that bioequivalence requirements are met, it is usually assumed that they are equally effective. The in vivo bioequivalence requirement impose a 90% confidence interval of the calculated averages of a bioequivalence metric, such as area under curve (AUC) and peak concentration C^max^ should fall within a prescribed bioequivalence limit, usually, 80%–125% for the ratio of the product averages. Biowaiver is well established for biopharmaceutics classification system (BCS) class I and has been extended to class III drugs when formulated as immediate release solid dosage form. This has been reasoned by an excellent correlation between in vitro–in vivo performances. In this case, difference factor (*f*_1_) and similarity factor (*f*_2_) can be used [10]. The latter factor measures the similarity in the percent of dissolution between the data points of the two tested products [11]. These methods, whether in vitro or in vivo, allow for certain differences between the brand and the generic product of up to 20% variation [12]. There is, however, a debate in the scientific community about whether or not such small differences matter [13,14]. 

Bacterial resistance is one of the most clinical and public health challenges facing the healthcare system [15]. Many reasons have been implicated for bacterial resistance associated with administering antibiotics, ranging from natural biological processes to misuse and inadequate diagnosis and treatment [16,17,18]. The frequent prescription of broad-spectrum antibiotics rather than prescribing more precise antibiotics and the inadequate dose and duration of treatment have been particularly embroiled. The subinhibitory concentrations resulting from the later effect can modulate the bacterial virulence [19,20]. A source of subinhibitory levels of antibiotic can be from the often neglected factor that generic drugs may not be in fact therapeutically equivalent to the branded antibiotic. So, the branded products maybe more effective than generics, a concept some are willing to accept [8,21,22,23]. Administering a generic antibiotic with poor drug release or subtherapeutic dose is not only ineffective, but can have the potential to expand bacterial resistance. 

The main purpose of this study was to investigate the in vitro bioequivalence of different combination products of amoxicillin trihydrate and potassium clavulanate present in immediate release tablets using Augmentin^®^ as the reference product. Other physical and chemical indicators such weight variation, chemical content, friability, and resistance to crushing were comprehensively analyzed and studied. 

## 2. Materials and Methods

### 2.1. Materials

Amoxicillin trihydrate raw material was generously donated by Neopharma pharmaceutical company (Abu Dhabi, United Arab Emirates), Augmentin^®^ (denoted as R), generic product 1 (denoted as T1), generic product 2 (denoted as T2), generic product 3 (denoted as T3), generic product 4 (denoted as T4), and generic product 5 (denoted as T5) are all 1 G products (875 mg of amoxicillin trihydrate +125 mg of potassium clavulanate) and were procured from local UAE pharmacies through Al Ain University of Science and Technology purchasing department. The details of the purchased products are given in Table 1.

Water HPLC Grade was purchased from Fisher Scientific U.K. (Leicestershire, U.K.), Methanol RS for HPLC isocratic and orthophosphoric acid 85% RPE were bought from Dasit Group, Carlo Erba Reagents (Val-de-Reuil, France), potassium phosphate monobasic extrapure AR was obtained from Sisco Research Laboratories (Mumbai, India). 

### 2.2. Methods

#### 2.2.1. Weight Variation

According to USP [24] and European Pharmacopoeia [25], weight variation can be used instead of content uniformity when the tablet contains 25 mg or more of an active drug or comprises 25% *w*/*w*. As such, weight variation test was applied to demonstrate the uniformity of the dosage units, since the claimed content of amoxicillin is 875 mg in each tablet. The test involved weighing 20 tablets individually using sensitive digital balance AUX 220 (±0.1 mg) (Shimadzu, Kyoto, Japan) from each product and then calculating the average mass. The percentage of individual tablet weight was then calculated to look at the individual deviations of the tablet weights from the average weight.

#### 2.2.2. Tablet Friability

The general USP method of tablet friability was followed [26]. 10 tablets from each product were dedusted, weighed, and then loaded into the clean drum of the friability apparatus TA 220 (Erweka, Heusenstamm, Germany). The tablets were tumbled in the rotating drum at the speed of 25 rpm for 4 min. The tablets were then observed for any cracks or broken parts, dedusted to remove any loose particles, and then weighed again to calculate the percentage of weight loss. 

#### 2.2.3. Resistance to Crushing of Tablets

Ten tablets from each product were tested using hardness tester TBH 225 TD (Erweka, Heusenstamm, Germany) which is also capable of recording the tablet’s length simultaneously. Each tablet was placed on a clean surface of the apparatus testing chamber and oriented so that the platens compressed parallel to the tablet longest axis. The mean of the resistance to crushing strength in Newtons and the standard deviation were calculated for each tested product. Additionally, the mean of the tablets’ length and standard deviation were calculated for each product. 

#### 2.2.4. Calibration Curve and HPLC Analysis of Amoxicillin

Stock solution (0.05% *w*/*w*) was prepared by dissolving an equivalent amount of 50 mg of amoxicillin from amoxicillin trihydrate in 100 mL of purified water. Dilutions were made appropriately to obtain a range of concentrations from 20 to 320 µg/mL for the preparation of calibration curves. An HPLC LC 20AD (Shimadzu, Kyoto, Japan) equipped with a high-precision gradient pump was used for the purpose of analysis at wavelength 220 nm by UV/Vis detector SPD-20A (Shimadzu, Kyoto, Japan). The column C18 with particle size 5 µm and a length of 250 mm and 4.6 mm internal diameter (Restek, Bellefonte, PA, USA) was rinsed with 70% methanol and 30% HPLC grade water at ambient temperature and purged with the mobile phase. The mobile phase was pumped at flow rate of 2 mL/min and consisted of 5% methanol and 95% of 0.05 M potassium dihydrogen orthophosphate, adjusted to pH 4.4 using orthophosphoric acid which was measured by the pH Meter Five Easy TM FE20 (Mettler-Toledo International Inc., Greifensee, Switzerland). All of the liquid preparations were filtered using polypropylene membrane filters with a 0.22 µm pore size (Restek, Beijing, China) under vacuum (Rocker Scientific, New Taipei City, Taiwan). Five different concentrations, 20 µL injection each, were used to prepare the calibration curve after ensuring reproducibility. The acquired data were processed and analyzed using LabSolutions software, Version 5.51 (Shimadzu Corporation, Kyoto, Japan) running on Windows 7 Professional (Microsoft Corporation, Redmond, WA, USA). The calibration curve was then used to determine the chemical content of amoxicillin in the tablets and also to determine the amounts of the drug dissolved from the dissolution experiment.

#### 2.2.5. Chemical Content of Amoxicillin

Twenty tablets from each product were weighed and powdered. An equivalent quantity of the powdered tablets containing 0.05 g of amoxicillin was dissolved in 80 mL of water, then sufficient water was added to produce 100 mL solution which was then filtered. A dilution was made to obtain an amoxicillin concentration equivalent to 100 µg/mL for HPLC analysis as described under the “calibration curve and HPLC analysis of amoxicillin” in order to determine the content of amoxicillin in the samples. This procedure was carried out in triplicate for each product. The mean percent of the chemical content based on labeled amoxicillin amount and the standard deviation were calculated for each tested product.

#### 2.2.6. Dissolution of Amoxicillin Tablets

Apparatus 2 for dissolution DT 820 (Erweka, Heusenstamm, Germany) was used at a pedal speed of 75 rpm. The dissolution medium consisted of purified water (900 mL) at 37 °C for testing each unit in each product (*n* = 12). Samples (10 mL) from the dissolution medium withdrawn were replaced with blank medium at the same temperature at the following times: 5, 10, 15, 20, 30, 45, 60, 80, and 120 min. The withdrawn samples were then filtered and prepared for analysis using the HPLC as described previously under the “calibration curve and HPLC analysis of amoxicillin” to determine the dissolved amount of amoxicillin. The calculated amounts included also any lost amoxicillin due to prior sample withdrawals. 

#### 2.2.7. Statistical Analysis

Statistical analyses were performed where appropriate using one-way analysis of variance (ANOVA) and *t*-test to compare between resistance to crushing strengths of tablets from different products. Significance difference was considered when *p* value < 0.05. Calculations, preparation of graphs and performing statistical analysis was made using Excel 2013, Microsoft Corporation, Redmond, WA, USA.

The model-independent mathematical approach developed by Moore and Flanner in 1996 using *f*_1_ and *f*_2_ were used to compare the dissolution profiles of the different generic amoxicillin to the branded amoxicillin tablets (R), the Augmentin^®^ [11]. *f*_1_ measures the relative difference between two curves and values up to 15% indicate little difference between the two curves. It is calculated as a percent (%) difference between the two curves according to the equation

(1) $$f_{1}=\left(\frac{\sum_{t=1}^{n}\left|R_{t}-T_{t}\right|}{\sum_{t=1}^{n}R_{t}}\right)\times 100$$

where *n* is the number of sampling times, *R_t_* is the cumulative percentage dissolved at each time point (*t*) of the reference, and *T_t_* is the cumulative percentage dissolved at each time point of the test product. *f*_2_ which is a measure of the similarity in the percentage of dissolution between two curves, was calculated from the equation

(2) $$f_{2}=50.Log\left\{{\left[1+\frac{1}{n}\sum_{t=1}^{n}{\left(R_{t}-T_{t}\right)}^{2}\right]}^{-0.5}\times 100\right\}$$

It is recommended that only one measurement should be considered after 85% dissolution of the products, since *f*_2_ is sensitive to the number of data points [27]. For the generic product to be considered similar to the original product, it is suggested that *f*_2_ should be between 50 to 100. Therefore, *f*_2_ values lower than 50 would indicate possible difference in the in vivo performance.

## 3. Results

### 3.1. Physical Quality of Amoxicillin Tablets

The results from weight variation, tablet friability, and resistance to crushing of tablets are given in Table 2. For tablet weight variation and friability tests, different products met the pharmacopeial standards. Weight variation did not exceed 3.5% and percent weight loss was zero for all tested products. The lowest average tablet weight was for the product T4 which differed from the highest average weight for product T5 by 137 mg. The mechanical strengths of tablets from different products were high as indicated by the friability and resistance to crushing force. The difference in the forces required to crush the tablets between highest and lowest mean values of R and T3 respectively was approximately 150 Newtons. ANOVA showed significant differences among the different products with regard to the resistance to crushing test (*p* < 0.001). These include significant differences from the reference product for products: T1, T2, and T3 (*p* < 0.001) and for T5 (*p* < 0.0013). Tablets from different products had close tablet lengths, except for the product tablets T5 which were approximately 10% longer.

### 3.2. Calibration Curve of Amoxicillin

**Figure 1:**
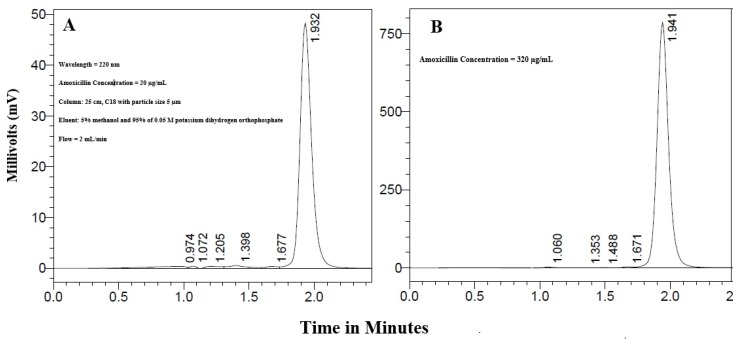
(**A**) Absorption peaks obtained for amoxicillin concentrations 20 µg/mL and (**B**) 320 µg/mL using HPLC.

Excellent correlation was obtained between amoxicillin concentrations in the range of 20 to 320 µg/mL and the measured AUC. Figure 1A,B show the peaks obtained for amoxicillin concentrations 20 and 320 µg/mL, respectively at about 1.935 min.

The calibration curve equation was AUC = 15395.6*x* + 4194.43 with coefficient of determination = 1.000.

### 3.3. Chemical Content of Amoxicillin Tablets

All tested tablets for amoxicillin content deviated by less than 5% from the labeled amount of the products (see Table 3). As such, the products would pass the USP chemical content requirement which has an allowable range from 90 to 120% [28].

### 3.4. Dissolution of Amoxicillin Tablets

**Figure 2:**
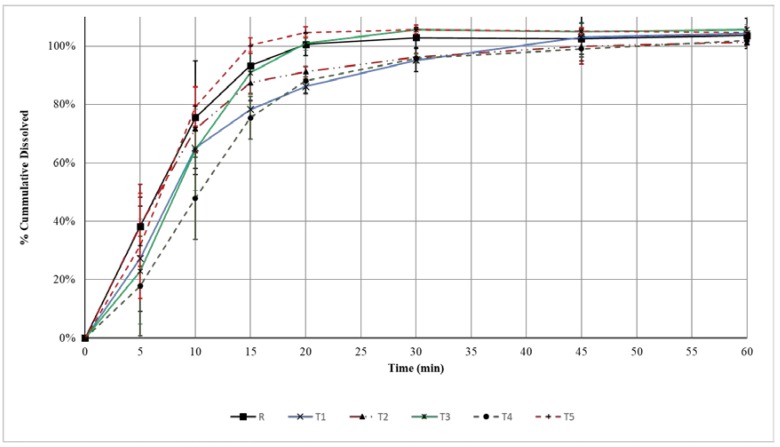
Dissolution profiles for amoxicillin trihydrate/potassium clavulanate tablets (1 G) from different brands available in local market. Error bars represent coefficient of variation (%).

The dissolution of amoxicillin from different products was rapid with the release of more than 85% of the labeled amount within 15 to 20 min (see Figure 2). As such, the products would pass the dissolution test standard in accordance with the USP requirement for amoxicillin tablets (i.e., Q ≥ 85% at 30 min) [28]. In fact R, T2, T3, and T5 are considered as very rapidly dissolving since at least 85% of labeled amoxicillin have dissolved in 15 min [29]. In these cases, similarity factor and difference factor calculations become unnecessary. The dissolution results also indicate that it was unnecessary to extend the dissolution time to 120 min as 60 min would have been sufficient to study the dissolution profiles adequately. The coefficient of variations for drug release at the time points 5 and 10 min were high but did not exceed 20% with any product. For other time points, these were less than 10%. 

For products T1 and T4, the calculated *f*_1_ exceeded the accepted limit of 15%, while for *f*_2_ the values are less than 50 (see Table 4). Therefore, both products fail to demonstrate in vitro bioequivalence requirements.

## 4. Discussion

Different amoxicillin tablets have shown satisfactory physical qualities including uniformity of tablet weights and mechanical strengths (Table 2). The latter is demonstrated by 0% loss from tablets following friability testing. With modern machinery and good formulation, it is possible to produce tablets within a desired weight range, which often supersedes the pharmacopeial minimum requirements.

The different resistance to crushing strengths that were obtained are relatively high in tablets, but this is maybe because of the tablet weights (over 1400 mg) and the tablet lengths (over 21 mm as measured concurrently in the resistance to crushing test), both of which will allow the tablet to absorb the applied breaking force. While tablets with high mechanical strength are desirable to overcome vibrations and subsequent handling such as packaging, transportation, and pushing the tablets out of their primary packaging, attention is paid as to not adversely affect drug’s dissolution. T3 product had a resistance to crushing strength equal to 66.6% of the reference product. This may influence how fast the drug will be released from the unit solid dosage form.

It was possible to obtain excellent correlation between concentrations and AUC in the tested range (Figure 1A,B). The flow rate used in the HPLC column 2 mL/min was in accordance with the USP. This is relatively high but was necessary for the adequate resolution of the amoxicillin (mol. wt. 365.4 Daltons) and clavulanate (mol. wt. 199.16 Daltons) peaks. The later peaks at about 1.39 min. This prevented overlap of the peaks and allowed accurate determination of the amoxicillin in the tablets.

Unlike BP, USP allows a wider range of amoxicillin in the tablets with as high as 120%. The upper limit in the BP is given as 105%. Despite the differences, all of the products would pass the test in accordance with the two pharmacopeia (Table 3). Although discouraged, the use of overages to compensate for drug degradation during manufacture or a product’s shelf life are apparent in the drug products (T1, T2, T3, T4, and T5), considering that the tested tablets were about half the way through their shelf lives at the times of experiments. Taking this into consideration, the values for % chemical contents, which are close to 100%, justify the use of the labeled amount as basis for calculating % of drug dissolved in the comparative dissolution experiments rather than based on the chemical content test.

The dissolution of tablets from different products (Figure 2) did not correlate well with the resistance to crushing strength. ANOVA indicated that tablets from different products had significantly different resistance to crushing strengths (*p* < 0.001). R and T3 which had the largest and lowest values for resistance to crushing respectively had more than 85% drug release in 15 min (Table 3). This is in agreement with the findings of others that not only the processing conditions, but also formulation factors, such as quality (Q1) and quantity (Q2) of disintegrants and diluents, play important roles in the tablet breaking forces and dissolution profiles [30,31]. 

Dissolution is considered an important tool to predict in vivo bioavailability and has been used to prove bioequivalence to allow interchangeability [14]. Very often FDA considers dissolution testing to be more discriminating than an in vivo test [32]. f_1_ and f_2_ have been used frequently for in vitro bioequivalence studies by comparing the dissolution profiles of two products. They have been adopted not only by the FDA Drug Evaluation and Research but also by Human Medicines Evaluation Unit of the European Agency for the Evaluation of Medicinal Products (EMEA) [33]. Amoxicillin belongs to the biopharmaceutics classification system group III indicating high solubility, but low permeability [34,35]. Combined with rapid dissolution, amoxicillin tablets can be eligible for the extension of biowaiver. In U.S.A., there are at least five generic products that have been approved as immediate release tablets containing amoxicillin trihydrate/potassium clavulanate tablets (1 G) with therapeutic equivalence evaluation code AB [36]. This code indicates the submission of adequate in vivo and/or in vitro evidence supporting bioequivalence. T1’s manufacturer has been recently granted approval of their amoxicillin trihydrate/potassium clavulanate tablets (1 G) in the U.S.A. Our in vitro dissolution study indicates that this locally marketed product may not be therapeutically equivalent with the reference product Augmentin^®^. A study published in 2012 used 12 amoxicillin products in the Americas found only three products to be in vitro equivalent to the reference comparator [37]. Another study found that the majority of generic brands of amoxicillin capsules in the Ethiopian market were deemed not interchangeable with the innovator brand following in vitro dissolution assessment [38]. This indicates that this problem is not limited to the local region.

The impact of prescribing T1 and T4 products on the health and safety of patients, including the development of bacterial resistance, needs further investigation. The current study did not cover tablet dissolution in different pH media and also included only the highest strength. Our dissolution study followed the recommended dissolution method suggested by the FDA, including dissolution medium, dissolution apparatus, and pedal speed and also adopted the USP method for amoxicillin/clavulanate potassium tablets monograph [28,39]. The combined clavulanate in the studied amoxicillin tablets enhances the latter’s effectiveness, but it was not studied. This is because the drug itself is not an effective antibiotic and several products exist without it. This study serves as an important example by which independent investigation can screen approved drug products and lead to the detection of inferior or poor quality ones. 

## 5. Conclusions

Independent investigation serves as an important additional tool to screen and test marketed products based on the criteria comparable to those authorizing their marketing, so they can be safe and effective. To that extent, this study is part of quality assurance that should be applied often to other important classes of drugs as well such as those used in the treatment of type 2 diabetes, hyperlipidemia, and hypertension. Different physical quality attributes indicate that amoxicillin trihydrate/potassium clavulanate tablets (1 G) available in the local market have satisfactory standards. The advances and sophistication of equipment used in the production of tablets contributes to the production of tablets with the desired weight uniformity and strength. This however is not enough to assure product’s in vivo performance. In vitro testing has indicated that two amoxicillin products in the UAE local market may not perform in accordance with their expected effectiveness and safety.

## Figures and Tables

**Table 1:** Amoxicillin trihydrate/potassium clavulanate tablets (1 G) of different brands purchased from local UAE market.

No.	Product	Manufacturing Country	Batch No.	Manufacturing Date (Month/Year)	Expiry Date (Month/Year)
1	R	U.K.	755708	11/2015	11/2017
2	T1	Jordan	6221	06/2015	06/2017
3	T2	UAE	XAG5001	11/2015	11/2017
4	T3	UAE	8948	12/2015	12/2017
5	T4	UAE	0245	05/2015	05/2018
6	T5	Saudi Arabia	155042	02/2015	02/2017

**Table 2:** Physical attributes of tested amoxicillin trihydrate/potassium clavulanate tablets (1 G) from different brands.

No.	Product	Average Weight (g)	Weight Variation Range (% from Average)	Tablet Friability (% Weight Loss)	Mean Resistance Force “N” ^$^ ± SD *	Mean Tablet’s Length in “mm” ± SD *
1	R	1.467	97.2%–102.0%	0.0%	453.1 ± 37.1	21.65 ± 0.03
2	T1	1.497	99.0%–101.4%	0.0%	343.6 ± 27.4 ^#^	21.68 ± 0.02
3	T2	1.534	97.1%–102.5%	0.0%	361.5 ± 35.3 ^#^	21.67 ± 0.03
4	T3	1.489	97.1%–103.5%	0.0%	301.6 ± 34.4 ^#^	21.44 ± 0.04
5	T4	1.453	98.9%–101.5%	0.0%	445.1 ± 27.7	21.64 ± 0.02
6	T5	1.590	97.7%–102.2%	0.0%	401.6 ± 21.2 ^#^	23.81 ± 0.03

* Standard deviation; ^$^ Newton; ^#^ significantly different from the reference product (*p* < 0.05).

**Table 3:** Mean percent of amoxicillin in each tested tablet product calculated on the basis of claimed amount ± standard deviation (SD).

Product	% Mean Chemical Content ± SD
R	99.42 ± 1.84
T1	100.58 ± 1.31
T2	104.31 ± 0.74
T3	102.88 ± 0.56
T4	102.42 ± 1.34
T5	103.88 ± 1.50

**Table 4:** Difference factor (*f*_1_), similarity factor (*f*_2_) and in vitro bioequivalence performances of five generic amoxicillin products in comparison to the reference product Augmentin^®^.

Generic Product	Difference Factor (*f*_1_)	Similarity Factor (*f*_2_)
T1	16.5%	44.5
T4	25.4%	34.6
